# Selective Histonedeacetylase Inhibitor M344 Intervenes in HIV-1 Latency through Increasing Histone Acetylation and Activation of NF-kappaB

**DOI:** 10.1371/journal.pone.0048832

**Published:** 2012-11-15

**Authors:** Hao Ying, Yuhao Zhang, Xin Zhou, Xiying Qu, Pengfei Wang, Sijie Liu, Daru Lu, Huanzhang Zhu

**Affiliations:** State Key Laboratory of Genetic Engineering, Institute of Genetics, School of Life Sciences, Fudan University, Shanghai, China; George Mason University, United States of America

## Abstract

**Background:**

Histone deacetylase (HDAC) inhibitors present an exciting new approach to activate HIV production from latently infected cells to potentially enhance elimination of these cells and achieve a cure. M344, a novel HDAC inhibitor, shows robust activity in a variety of cancer cells and relatively low toxicity compared to trichostatin A (TSA). However, little is known about the effects and action mechanism of M344 in inducing HIV expression in latently infected cells.

**Methodology/Principal Findings:**

Using the Jurkat T cell model of HIV latency, we demonstrate that M344 effectively reactivates HIV-1 gene expression in latently infected cells. Moreover, M344-mediated activation of the latent HIV LTR can be strongly inhibited by a NF-κB inhibitor aspirin. We further show that M344 acts by increasing the acetylation of histone H3 and histone H4 at the nucleosome 1 (nuc-1) site of the HIV-1 long terminal repeat (LTR) and by inducing NF-κB p65 nuclear translocation and direct RelA DNA binding at the nuc-1 region of the HIV-1 LTR. We also found that M344 synergized with prostratin to activate the HIV-1 LTR promoter in latently infected cells.

**Conclusions/Significance:**

These results suggest the potential of M344 in anti-latency therapies and an important role for histone modifications and NF-κB transcription factors in regulating HIV-1 LTR gene expression.

## Introduction

Highly active antiretroviral therapy (HAART) has succeeded in lowering human immunodeficiency virus type 1 (HIV-1) levels in most patients, in some cases to undetectable levels. However, this therapy alone cannot completely eradicate the virus [1], [2]. Many studies have shown that this is most likely because of a stable population of latently infected CD4^+^ T cells, which cannot be elimated by HAART on its own [3]–[5]. The small pool of latently infected cells that is present in each infected individual functions as a reservoir for the virus. Because of its slow decay rate [6]–[10], this reservoir is now considered to be the main barrier to viral eradication via current antiretroviral drugs [6], [9], [10].

Much progress has recently been made to elucidate the molecular mechanisms underlying HIV-1 proviral latency, which is intimately tied to HIV-1 transcription level [11]–[13]. Several factors contribute to the transcriptional silencing of integrated HIV-1 proviruses. The first is the site of proviral integration into the host cell genome and the cellular chromatin environment at this site [14]–[16]. The second mechanism involves the epigenetic silencing by post-transcriptional modifications (e.g., hypoacetylation or trimethylation) on histones that are key components of nucleosome and capable to modulate the chromatin structure [16]–[18]. The third mechanism involves the ability of host cell factors to restrict HIV-1. Transcription factors such as yin and yang 1 (YY1) and late SV40 factor repress HIV-1 replication in infected CD4^+^ T cells by recruiting HDAC1 to the repressor complex sequence located at nucleotides –10 to +27 in the LTR [19]–[20]. Other host transcription factors, such as NF-κB subunit p50 homodimers and C-promoter binding factor 1, can also recruit HDACs to the LTR and inhibit viral transcription similarly to YY1 in several cell lines [21], [22]. The fourth mechanism involves the microRNAs (miRNAs) and RNA interference (RNAi). It has been shown that cellular miRNAs may inhibit HIV-1 gene expression by interfering with histone acetylation [23]. Some miRNAs have also been shown to directly target HIV-1 messenger RNA (mRNA), suppressing the viral gene expression. Five cellular miRNAs in particular have been found to target the 3′ end of HIV-1 mRNAs in resting CD4^+^ T cells. These miRNAs have been shown to be upregulated in resting CD4^+^ T cells relative to activated CD4^+^ T cells [24], [25], further linking them to latency. The fifth mechanism involves the inefficient elongation of HIV-1 transcripts, owing to the absence of the viral protein Tat and Tat-associated viral factors [26]–[29].

A group of antilatency therapeutic strategies nicknamed “shock and kill” was proposed based on this molecular understanding of HIV-1 latency [30], [31].These strategies are based on activation of HIV-1 expression in latently infected cells by stimuli, either triggering virus-mediated cell lysis or rendering the cells susceptible to drugs or antibodies [32], [33]. Certain stimulants, such as interleukin (IL)-2 and anti-CD3 antibodies, already show potency in this regard [34], [35]. Margolis lab and Verdin lab had reported that HDAC inhibition trichostatin A (TSA) can induce HIV-1 expression in cell line models of latency [36], [37], respectively. The Carine Van Lint lab has also demonstrated a strong synergistic activation of HIV-1 promoter activity by the HDAC inhibitors TSA and the NF-κB inducer tumor necrosis factor-α (TNF-α) in the postintegration latency model cell line U1 [38]–[40], suggesting that combinations of two independent factors (NF-κB and chromatin) involved in HIV-1 reactivation from latency might be potent tools to decrease the pool of latently-infected cells. However, because of their toxicity, therapeutic use of TNF-α and TSA is not possible. At present, various new activators have also been described, including interleukin (IL)-7[41]–[43], prostratin [44]–[46], valproic acid [47], [48], suberoylanilide hydroxamic acid (SAHA) [49], apicidin [50], metacept-1 and metacept-3 [51], buthionine sulfoximine (BSO) [52], hexamethylene bisacetamide (HMBA) [53] and BIX0129 [54]. Interestingly, these compounds stimulate HIV replication, and their effects on the T cell activation appear limited.

M344, are stable analogues of TSA with substrate selectivity for HDAC6 [55]. M344 has been reported to inhibit the growth and induce apoptosis in a variety of cancer cells [56], [57]. The drug is considered to be a novel HDAC inhibitor with robust activity and relatively low toxicity compared to TSA [56], [57]. This study was aimed at investigating the ability of the selective HDAC inhibitor M344 to induce expression of HIV-1 in latently infected cells, the molecular mechanisms of M344 reactivation of HIV-1, and the effect of M344 combined treatment with others activators on HIV-1 transcriptional initiation. Our results confirmed that M344 can induce the reactivation of latent HIV-1 transcription, and this was associated with significant change in histone H3 and H4 acetylation levels at the HIV-1 LTR promoter and inducing NF-κB p65 nuclear translocation and direct RelA DNA binding at the nuc-1 region of HIV-1 LTR. We also found that M344 synergized with prostratin to activate the HIV-1 LTR promoter in latently infected cells. Our results suggest that histone modifications and NF-κB transcription factors play a prominent role in regulating HIV-1 LTR gene expression and that HDAC6-selective inhibitor M344 may be a candidate for antilatency therapies.

## Results

### M344 Potently Activates Latent HIV-1 Replication

**Figure 1:**
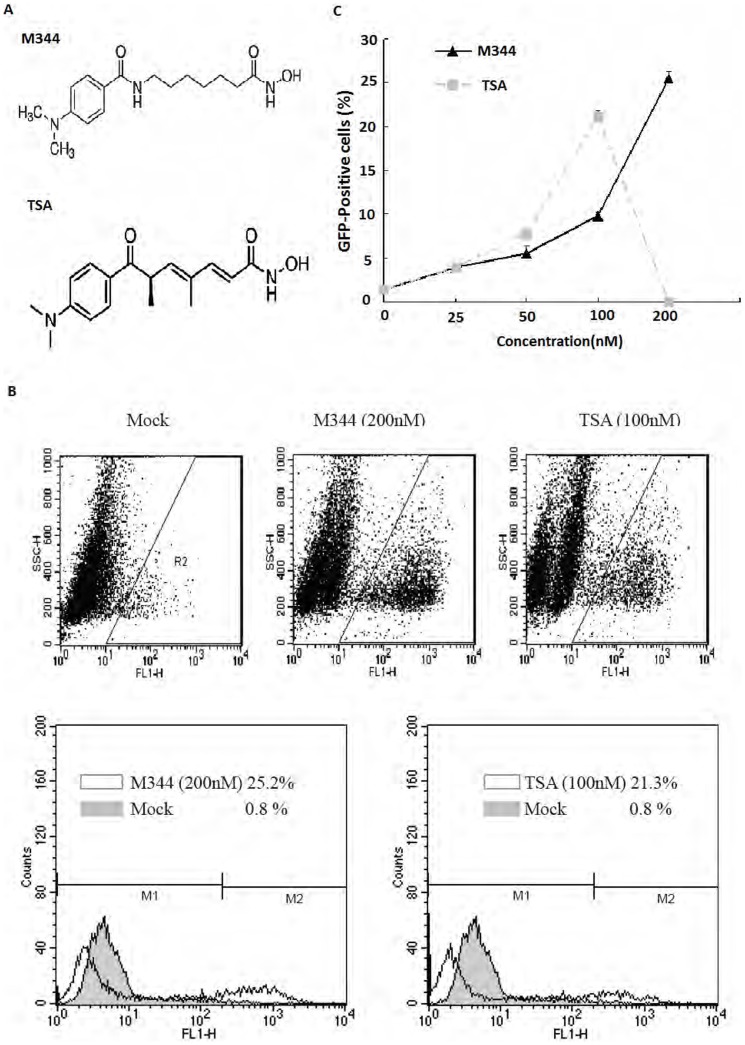
Reactivation of latent HIV-1 in latently infected cells by M344 and TSA. (A) The structure of M344 and TSA. (B) J-Lat clones A7 cells, which have an integrated GFP/Tat construct under control of the HIV-1 LTR, were treated with M344 or TSA at the indicated concentrations for 72 hours. The percentage of cells expressing GFP was measured by flow cytometry, to determine the level of HIV-1 expression. Results are presented as fluorescence histograms. (C) Dose-dependent effects of M344 on HIV-1 production. Data represent the means±standard deviations of three independent experiments.

The structure of M344 and TSA are shown in Figure 1A. To investigate whether M344 induces expression of HIV-1 in latently infected cells, J-Lat clones A7 cells, which are latently infected Jurkat cells encoding the green florescence protein (GFP) under control of the HIV-1 LTR as a marker of HIV-1 LTR expression, were treated with M344 or TSA at different concentrations for 72 hours, and then the percentage of GFP-expressing cells was measured by flow cytometry. Three days after treatment with 200 nM M344, expression of HIV-1 activity was obtained, and the percentage of GFP-expressing cells was found to be as high as 25.2% more than those subjected to mock treatment (Fig. 1B). TSA (100 nM) induced GFP-expressing cells up to 21.3% (Fig. 1B). As shown in Figure 1C, addition of nanomolar concentrations of M344 to the culture medium for 3 days increased the percentage of GFP-expressing cells by 4–25-fold over background levels. However, induction by TSA was similar to that observed following M344 treatment. TSA was found to be very toxic to A7 cells at routinely used concentrations of 200 nM, as analyzed by proiodide staining (Fig.S1). These results were confirmed visually by fluorescence microscopy (data not shown). A summary of various HDAC inhibitors at their indicated concentrations to stimulate the expression and activity of HIV-1 LTR in A7 cells is shown in Figure 1C. These results confirmed that M344 induced HIV-1 LTR reactivation, indicating M344’s effects on HIV-1 production to be dose-dependent.

**Figure 2:**
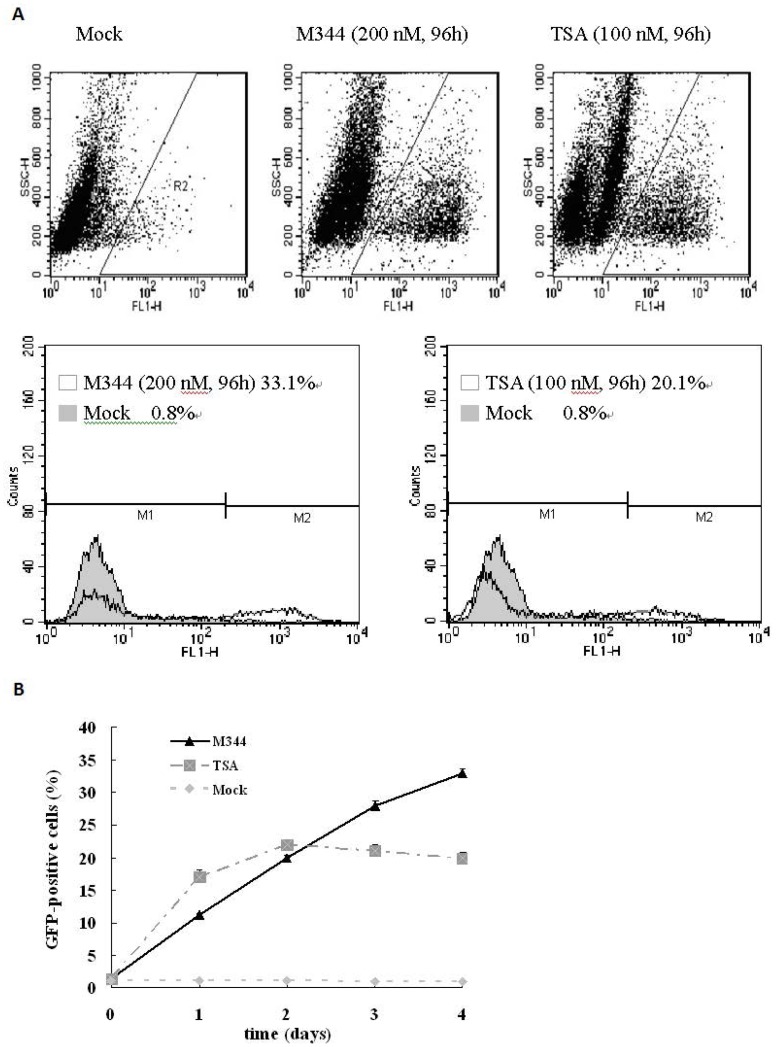
Kinetic analysis of reactivation of latent HIV-1 in latently infected cells by M344 and TSA. (A) J-Lat clones A7 cells were mock treated or treated with M344 (200 nM) or TSA (100 nM) at the indicated times. GFP expression was monitored in gated live cells at 1, 2, 3, and 4 days by standard flow cytometric techniques. Results are presented as fluorescence histograms. (B) Time-dependent effects of M344 on HIV-1 production. Data are expressed as percentage of cells becoming GFP-positive, and represent the means standard deviations of three independent experiments.

To analyze the kinetics of HIV-1 LTR expression induction by M344 concentrations optimal for eliminating latently infected cells, we performed a kinetics experiment in which J-Lat clones A7 cells were grown for 1–4 days with or without M344 (200 nM) or TSA (100 nM). At each time point, GFP-expressing cells were assayed by flow cytometry (Fig.2A). As shown in Figure 2, after the J-Lat clones A7 cells were treated with M344, the percentage of GFP-expressing cells increased over time. Four days after treatment, we observed that the percentage of GFP-expressing cells was as high as 33.1% for M344 (Fig. 2A). The kinetics of HIV LTR expression induction by TSA (100 nM) show a rapid rise for the first 2 days then a plateau by day 4 (Fig. 2B). These result were also confirmed visually by fluorescence microscopy (data not shown), indicating M344’s effects on HIV-1 production to be time-dependent.

To examine whether similar results could be obtained in other latently infected T cells, we therefore established HIV-1 latently infected cells C11. The result also showed M344 can potently activate latent HIV-1 replication (data not shown).

### Synergistic Reactivation of Latent HIV-1 Promoter by M344 Combination with Prostratin

**Figure 3:**
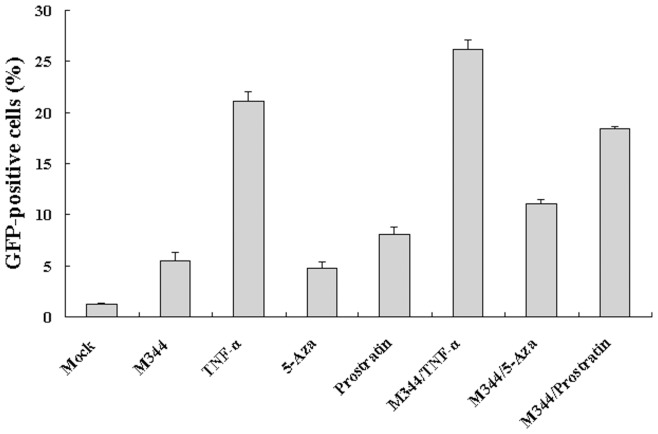
Synergistic activation of HIV-1 promoter by M344 and TNF-α, 5-Aza and prostratin in latently infected cells. J-Lat clones A7 cells were mock treated or treated with M344 (50 nM), TNF-α (10 ng/ml), 5-Aza (500 nM), prostratin (100 nM), M344/TNF-α, M344/5-Aza or M344/prostratin. The effects of synergistic activation of HIV-1 promoter were determined by quantifying the GFP-positive cells using flow cytometry 72 hours after treatment. Results are presented as fluorescence histograms. Summary of synergistic activation assays are presented as histograms. Data represent the means standard deviations of three independent experiments.

To assess whether M344 synergistically reactivates the HIV-1 promoter in J-Lat clones A7 cells when combined with TNF-α, 5-Aza, or prostratin, J-Lat clones A7 cells were treated with or without M344 alone (50 nM), TNF-α alone (10 ng/ml), 5-Aza alone (100 nM), prostratin alone (100 nM), or M344 (50 nM)/TNF-α (10 ng/ml), M344 (50 nM)/5-Aza (100 nM), or M344 (50 nM)/prostratin (100 nM) for 48 hours, respectively. Two activators synergize when their combination produces a percentage of GFP-expressing cells that is more than the sum of the effects produced by the individual activators. As shown in Figure 3, in the absence of stimulation, J-Lat clones A7 cells expressed almost no GFP, indicating a blockage of viral transcription. Stimulation of the J-Lat clones A7 cells with M344 alone or 5-Aza alone or TNF-α alone or prostratin alone induced GFP expression in a small proportion of cells (5.5%, 4.8%, 21.1%, and 8.1% of cells, respectively) (Fig. 3), indicating that following treatment with M344 alone, 5-Aza alone, TNF-α alone, or prostratin alone, the percentage of GFP-expressing cells as a quantitative marker of HIV-1 transcription in each group was higher than that of the mock group. Importantly, the proportion of J-Lat clones A7 cells displaying GFP epifluorescence was strongly and synergistically increased by M344/prostratin (18.4%), not by the M344/5-Aza (11.1%) or the M344/TNF-α combinations (26.2%) (Fig.3). These results indicated that, in the J-Lat clones A7 cells, the M344 combinatorial treatment with prostratin caused the synergistic recruitment of unresponsive J-Lat clones A7 cells into the expressing cell population. We measured cotreatmenttwo activators viability on HEK 293 cells at concentrations used to measure synergistic reactivation. There were no significant differences in cells viability between cotreatment two activators and individual activators (Fig. S2).

### M344 with Low Toxicity Compared to TSA *in vitro*

**Figure 4:**
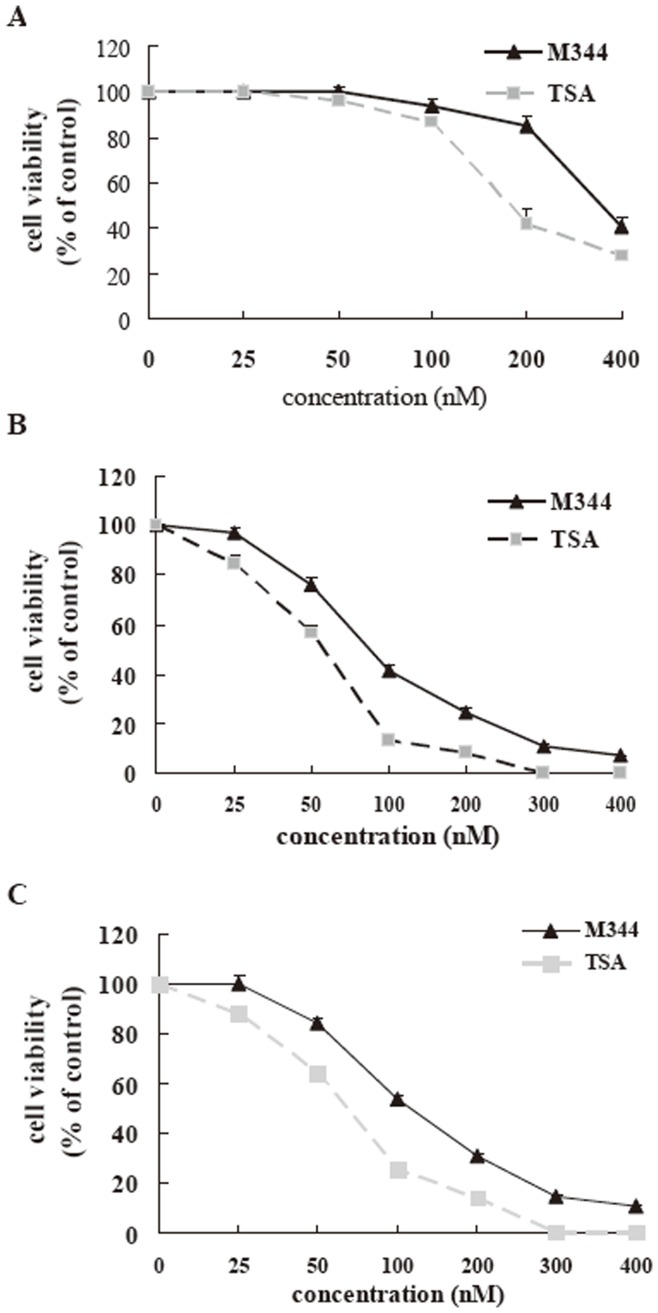
Summary of cell viability assays using M344 and TSA. 293- Human Embryonic Kidney (A), J-Lat clones A7 cells (B) and Jurkat T cells (C) were treated with M344 or TSA at the indicated concentrations for 48 hours, and measured by the MTT method. Results are presented as a percentage of the O.D. (P = 550) of untreated controls subtracted for background. Data represent the means±standard deviations of three independent experiments.

To measure viability, HEK 293 cells, J-Lat clones A7 cells, Jurkat T cells and human primary CD4**^+^** T cells were treated with or without M344 or TSA for 48 hours, and then the cells were subjected to an MTT assay. We found a significant correlation between the concentration of HDAC inhibitors and MTT expression in the HEK 293, J-Lat clones A7 cells and Jurkat T cells (Fig.4). The CC50 in the HEK 293, J-Lat clones A7 cells and Jurkat T cells for M344 was 352, 88, and 124 nM, respectively. The CC50 in the HEK 293, J-Lat clones A7 cells and Jurkat T cells for TSA was 181, 61, and 73 nM, respectively. The low toxicity was also observed in the primary CD4**^+^** T cells following incubation with M344 at the same concentrations as TSA (Fig.S3). These results indicated that M344 is low toxicity at its active concentration, and further evaluation of cytotoxicity in animal models will be a critical step in the clinical development of these compounds.

### M344 Increases Acetylation of Histone at the nuc-1 Region of HIV-1 LTR

**Figure 5:**
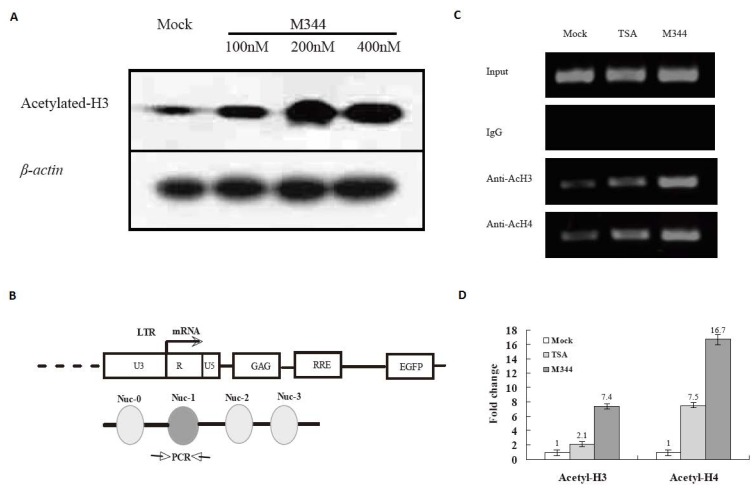
Histone acetylation modification at HIV LTR promoter. (A) Western blot detection of acetylated histone H3 levels in latently infected cells treatment with M344. J-Lat clones A7 cells were mock treated or treated with M344 (100 nM, 200 nM, 400 nM), and cell lysates were harvested after 8 hours. Western blot analysis was performed with antibodies acetylated histone H3. The amount of protein was normalized by comparison to levels of β-actin. (B) Diagram shows the positions of nucleosomes bound to the HIV-1 LTR and the location of primer used for PCR amplification in the ChIP assay. (C)Chromatin fragments from J-Lat clones A7 cells cultured for 4 hours with or without M344 (200 nM) or TSA (200 nM) were immunoprecipitated with antibody to acetylated histones H3 (AcH3) and H4 (AcH4) or control normal rabbit serum (IgG). PCR primers for the LTR promoter were used to amplify the DNA isolated from the immunoprecipitated chromatin as described in Materials and Methods. (D) Each ChIP experiment was repeated three times to confirm reproducibility of results and real-time quantitation of the fold change relative to untreated control is shown.

To determine whether M344 induces acetylation of histones, we treated J-Lat clones A7 cells with 100 nM, 200 nM, and 400 nM M344 for 8 hours and performed immunoblot analysis using antibodies to acetylated histones H3. As shown in Figure 5A, exposure of cells to 100–400 nM M344 results in a significant increase in acetylated histone.

In order to determine if the reactivation of latent HIV-1 in response to M344 was due to hyperacetylation of the HIV-1LTR promoter region, ChIP assays were performed. Chromatin fragments from J-Lat clones A7 cells cultured with or without M344 (200 nM) or TSA (200 nM) for 4 hours were immunoprecipitated with antibodies to acetylated histones H3 or H4 or rabbit preimmune IgG. DNA from the immunoprecipitates was isolated, and PCR was performed using HIV promoter primers spanning the nuc-1 region of LTR (Fig. 5B). We observed that the amounts of acetylated histone proteins, Ac-H3 and Ac-H4, bound to the core promoter region within HIV-1 LTR were increased by the treatment of cells with M344 or TSA (Fig. 5C). Normal IgG control showed no specific 190-bp fragment (Fig.5C). The percentage of input for each immunoprecipitation was calculated and the relative fold occupancy of acetylated histones reported. Fold increase in immunoprecipitation over mock antibody immunoprecipitation is shown in Figure 5 D. We found that M344 treatment increased the acetylation of H3 (7.4-fold) and H4 (16.7-fold) within nuc-1 in J-Lat clones A7 cells relative to mock treatment, while TSA treatment increased the acetylation of H3 (2.1 -fold) and H4 (7.5-fold) within nuc-1.

### HDAC6 is Localized in Both the Nucleus and the Cytoplasm in J-Lat Clones A7 Cells but not Recruited to the HIV-1 LTR Promoter

**Figure 6:**
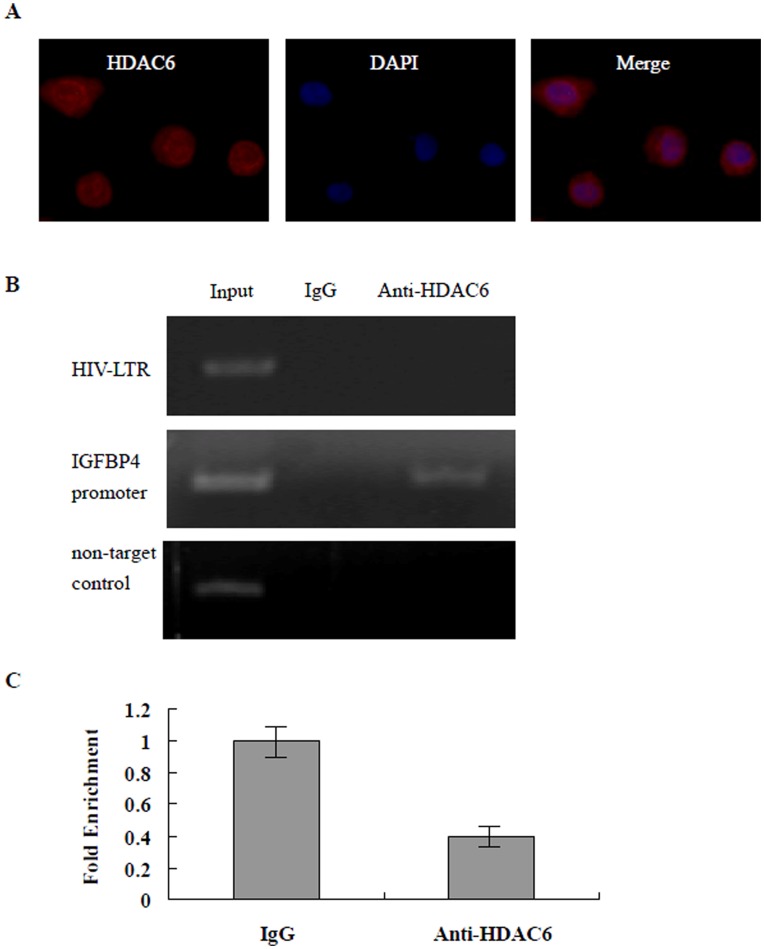
Subcellular localization and ChIP analysis of HADC6 in the J-Lat clones A7 cells model of latency. (A) HDAC6 is present in the nucleus and cytoplasm of J-Lat clones A7 cells. Subcellular localization of HADC6 was assessed by immunofluorescence analysis with a rabbit polyclonal anti-HADC6 IgG antibody and a goat anti-rabbit IgG antibody coupled with Alexa-555 (red color). DAPI staining was used to determine the nuclear region and to assess gross cell morphology. (B) ChIP analysis of HDAC6 occupancy at the HIV-1 LTR promoter in J-Lat clones A7 cells. IGFBP4 promoter was used as a positive-control region of DNA in J-Lat clones A7 cells to verify the ability of HDAC6 antibodies to work in ChIP assays, and rabbit IgG serum was used as a negative control. Chromatin fragments from J-Lat clones A7 cells were immunoprecipitated with antibody to HDAC 6 or control normal rabbit serum (IgG). PCR primers for the LTR promoter or IGFBP4 promoter or IGFBP4 non-targeting DNA were used to amplify the DNA isolated from the immunoprecipitated chromatin as described in Materials and Methods. (C)Each ChIP experiment was repeated three times to confirm reproducibility of results and the values represent the enrichment of LTR DNA over the IgG negative control as determined by quantitative PCR.

Recently, it has been reported that HDAC1, HDAC2, and HDAC3 can be recruited to a site at the HIV LTR and may play a role in the repression of LTR expression [21], [22]. Our data show that HDAC6-selective inhibitor M344 was effective in inducing HIV-1 LTR expression in J-Lat clones A7 cells. For this reason we further investigated the association of HDAC6 with the HIV-1 LTR promoter during latency in J-Lat clones A7 cells. First, we determined the cellular localization of HDAC6 in A7 cells by immunohistochemistry using rabbit polyclonal antibodies against HDAC6. As shown in Figure 6A, HDAC6 was detected in both the nucleus and the cytoplasm of J-Lat clones A7 cells. Thus, J-Lat clones A7 cells are a reasonable model cell line to evaluate HDAC recruitment to the integrated HIV-1 LTR. Next, we investigated the association of HDAC6 with the HIV-1 LTR promoter during latency by ChIP assay using antibodies directed against HDAC6. We used a sonication protocol that generates chromatin fragments about 1000 bp. Fragments of this size range can contain more than one nucleosome and therefore allowed us to examine changes in histone acetylation over a larger region of the LTR. In these experiments, the IGFBP4 promoter was used as a positive-control region to verify the ability of HDAC6 antibodies to work in ChIP assays [58]. As shown in Figure 6B, normal rabbit serum generated no PCR products after immunoprecipitation. Anti-HDAC6 antibodies were able to immunoprecipitate the IGFBP4 promoter region but not non-target DNA (Fig. 6B). In contrast, no amplification was observed when DNA immunoprecipitated by anti-HDAC6 antibody was used as a template for two different pairs of primers on the HIV-1 LTR promoter (Fig. 6B). Quantitative PCR yielded no significant amount of HIV-1 LTR DNA enrichment was detected relative to the IgG negative control (Fig. 6C). Taken together, these results indicate that HDAC6 is not recruited to the HIV-1 LTR promoter in the J-Lat clones A7 cell model of latency.

### M344 Activates the HIV-1 LTR Through Induction of NF-κB

**Figure 7:**
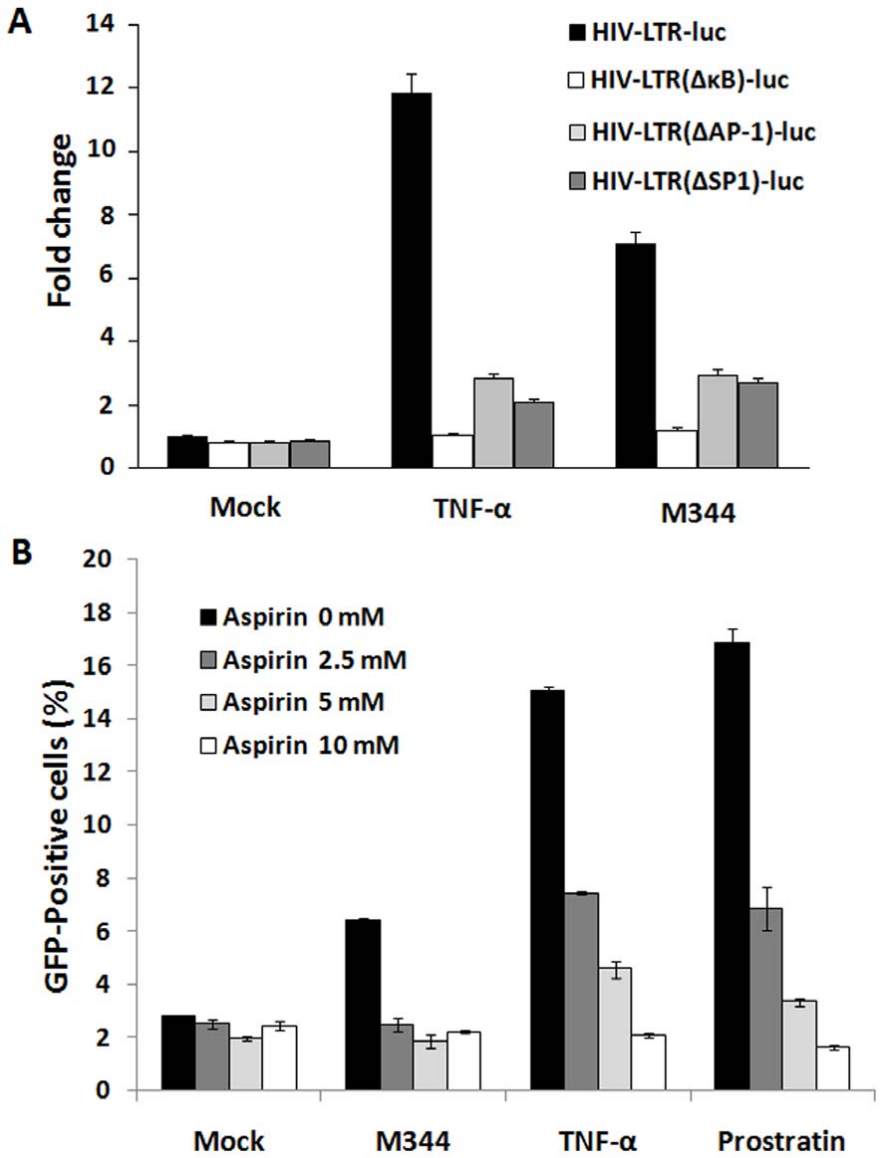
M344 activates the HIV-1 LTR through induction of NF-κB. (A) J-Lat clones A7 cells were transfected with HIV1-LTR luc, HIV1-LTRΔκB luc, HIV1-LTRΔAP-1luc, and HIV1-LTRΔSp1luc. At 24 hours posttransfection, the cells were treated or mock treated with M344 (200 nM) or TNF-α (10 ng/ml). Luciferase activity was measured after 24 hours of stimulation. The error bars indicate standard deviation. (B) J-Lat clones A7 cells were pretreated with various concentrations of (0, 2.5, 5 and 10 mM) aspirin for 3 hours and subsequently treated with M344 (100 nM) or TNF-α (10 ng/mL) or prostratin (100 nM) or control medium for 24 hours. The percentage of GFP+ cells (y-axis) in M344 or TNF-α stimulated cells in either the absence or the presence of the chemical inhibitors was measured by flow cytometry. Data represent the means±standard deviations of three independent experiments.

Previous studies show that M344 is a potent activator of NF-κB transcription factor [59]. We thus explored whether M344 activates the HIV-1 LTR through induction of NF-κB signaling pathway in J-Lat clones A7 cells. The HIV-1 LTR contains binding sites for several inducible transcription factors, including NF-κB, AP-1, and Sp1. To assess the role of NF-κB factors in M344 activation of the HIV LTR, J-Lat clones A7 cells were transfected with luciferase reporter plasmids containing either the wild type HIV-1 LTR, the LTR lacking the two κB enhancers, the LTR lacking the AP-1 enhancers, or the LTR lacking the Sp1 enhancers. M344 induced 7-fold stimulation of the HIV-LTR-Luc reporter relative to mock controls but failed to activate the HIV-LTRΔκB-Luc reporter (Fig.7A). Additionally, M344 induced about 3-fold stimulation of the HIV-LTRΔAP-1-Luc and HIV-LTRΔSp1-Luc reporters (Fig. 7A), indicating that neither AP-1 nor Sp1 is required for HIV LTR responsiveness to M344. Together, these findings support a central role for NF-κB induction in M344-mediated activation of the latent HIV LTR and exclude a necessary role of AP-1and Sp1.

To further confirm directly the role of NF-κB factors in M344 activation of the HIV LTR, J-Lat clones A7 cells were pretreated aspirin, which can inhibit TNF-α-induced activation of NF-κB [60], [61], and subsequently treated with M344 (100 nM) or TNF-α(10 ng/mL) or prostratin (100 nM) or control medium. Aspirin pretreatment not only inhibit TNF-α and prostratin-induced GFP expression in a dose-dependent manner, but also strongly inhibit GFP expression induced by M344 at the concentrations tested (Fig.7B and Fig. S4), further implicating a NF-κB-dependent signaling step in this response.

### M344 induces NF-κB Nuclear Translocation and Direct RelA DNA Binding at the nuc-1 Region of HIV-1 LTR

**Figure 8:**
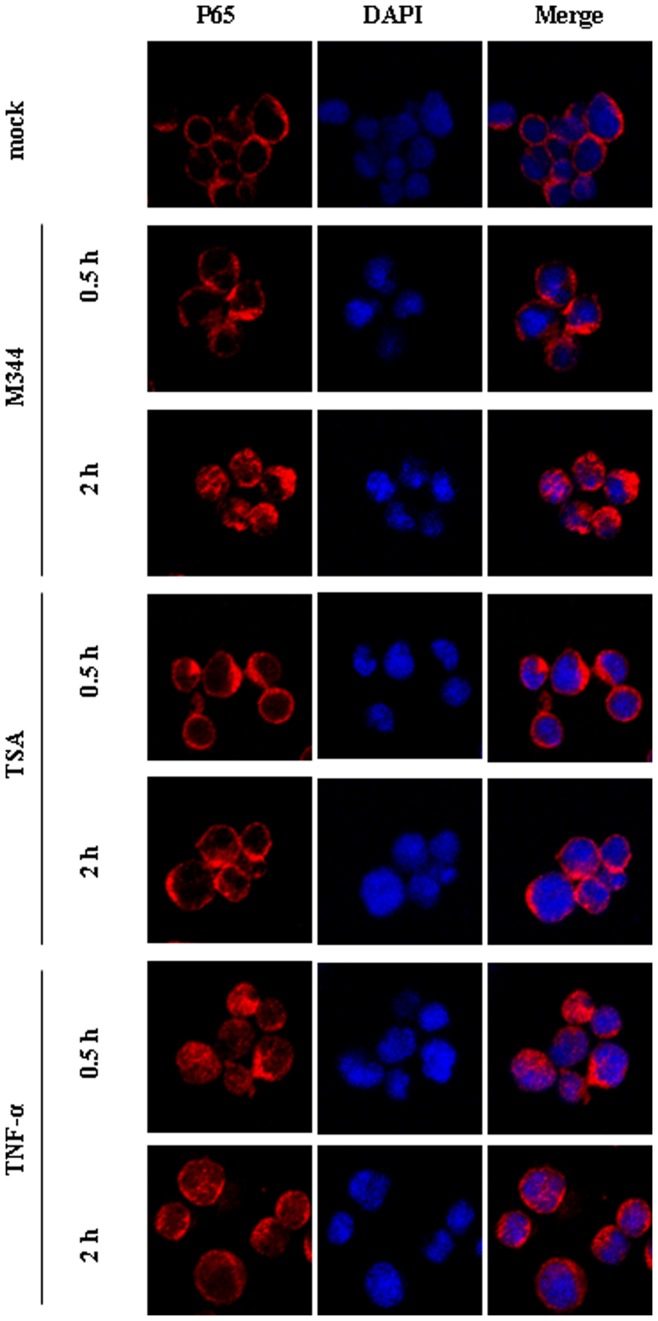
Subcellular localization of p65. Immunofluorescence analysis of the p65 protein in J-Lat clones A7 cells mock treated or treated with M344, or TNF or TSA for 30 minutes or 2 hours. Subcellular localization of p65 was determined via indirect immunofluorescence employing rabbit polyclonal anti-p65 and goat anti-rabbit antibody coupled to Alexa-555. DAPI staining was used to determine the region of nuclei and to assess gross cell morphology.

To assess whether M344 stimulation provided sufficient stimulus for RelA nuclear translocation and DNA binding, we studied the effect of M344 on the subcellular distribution of p65. We monitored the localization of the endogenous p65 protein during stimulation with M344, TSA, TNF-α by confocal microscopy (Fig. 8). In unstimulated J-Lat clones A7 cells, p65 was localized predominantly in the cytoplasmic compartment. Treatment with M344 for 30 minutes did not alter this subcellular distribution. A 2-hour treatment with M344 caused a translocation of p65 into the nucleus. Treatment with TSA for 30 minutes or 2 hours did not alter this subcellular distribution. Treatment with TNF-α led after 30 minutes to the migration of p65 to the nucleus. Following 2 hours of treatment with TNF-α, we observed the return of the nuclear p65 to the cytoplasm. These results indicate that M344 can induce NF-κB nuclear translocation.

**Figure 9:**
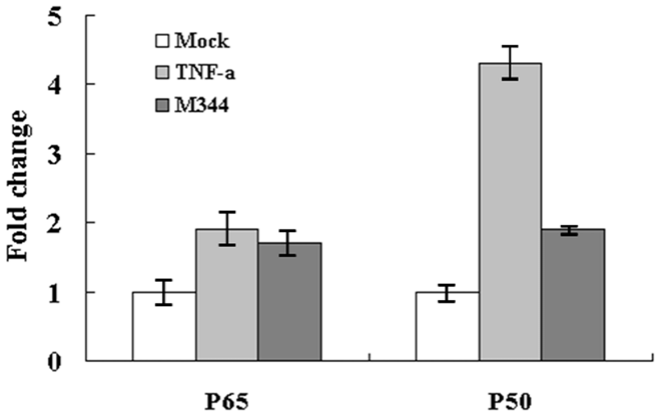
M344 induces RelA recruitment to the latent HIV-1 LTR. J-Lat clones A7 were stimulated with M344 (200 nM) or TNF-α(10 ng/ml) for 4 hours, respectively. Chromatin immunoprecipitation assays were performed using anti-p65 or anti-p50 antibodies or rabbit preimmune IgG and probed for the HIV LTR DNA sequences spanning the κB enhancer or for nonspecific control β-actin. Each ChIP experiment was repeated three times to confirm reproducibility of results and real-time quantitation of the fold change relative to untreated control is shown.

To investigate whether RelA is directly recruited to the HIV LTR in vivo following M344 stimulation, chromatin immunoprecipitation assays were performed. J-Lat clones A7 were treated with M344 or TNF-α, respectively, for 4 hours. Next, the DNA from the cross-linked cells was fragmented via digestion with micrococcal nuclease and sonication. Lysates were immunoprecipitated with anti-p65 or anti-p50 antibodies or rabbit preimmune IgG. These precipitates were then investigated for fragments of HIV-LTR sequences. In the absence of stimulation, samples immunoprecipitated with anti-p65 amplified low to undetectable levels of HIV LTR κB binding site DNA. Following stimulation M344 or TNF-α, anti-p65 immunoprecipitated samples from both J-Lat clones A7 amplified significant quantities of HIV LTR DNA. In contrast, stimulated and unstimulated samples with M344 or TNF-α immunoprecipitated with NF-κB p50 antibodies were enriched in HIV LTR DNA. Fold increase in immunoprecipitation over mock antibody immunoprecipitation is shown in Figure 9. In addition, these samples did not amplify β-actin negative controls beyond background levels (data not shown), demonstrating specificity of the DNA immunoprecipitation.

## Discussion

The latently HIV-1-infected monocytic cell lines U1 and ACH2 have long been used to study cellular models of postintegration latency [62], [63]. However, mutations in Tat (U1) or in its RNA target TAR (ACH2) have been demonstrated to be causative of the latent phenotype of the proviruses integrated in these two cell lines. More recently established J-Lat cells, developed with an HIV-1-based vector containing an intact Tat/TAR axis [64], [65], were selected for a lack of GFP expression under basal conditions. However, upon appropriate stimulation, such as with the NF-κB inducer, TNF-α, or the HDAC inhibitor TSA, viral transcription is activated and viral expression can be measured by cytometric detection of GFP epifluorescence [64], [65]. When the stimuli are removed, the cells return to their latent state. J-Lat cells represent valuable tools for studying HIV transcriptional silencing mechanism and for screening small molecules that can reactivate latent HIV. For this reason, we chose to employ J-Lat Tat-GFP Clone A7 cells in this experiment.

Previous studies have demonstrated the transcriptional activation of the HIV-1 promoter in response to HDAC inhibitors such as TSA, trapoxin (TPX), and valproic acid (VPA) [40], [48], [66]. However, the fact that these inhibitors are not class-specific, and interrupt many cellular parthways, their toxicity issues have raised a fair amount of concerns in the field [67]–[69]. Class I HDACs, such as HDAC1-3 and 8, are predominantly nuclear enzymes [70]. Class II HDACs include HDAC4-7, 9, and 10, which transport between the nucleus and the cytoplasm [71], [72]. Like in many such cases, the choice of an inhibitor most specific to the individual HDACs relevant to HIV-1 LTR regulation may lead to better targeting and reduced toxicity. As global HDAC inhibition may have adverse effects on host cells, we investigated the ability of M344, a potent selective factor for HDAC6 inhibitors, to induce LTR expression in A7 cells and the effects of its on the viability of the cell line *in vitro*. As a reference standard for the comparison of results, we used TSA, a non-specific inhibitor of both classes of HDACs when used in upper-nanomolar concentrations. Our results illustrate that M344 was not only shown to be effective in inducing HIV-1 LTR expression in A7 cells, but also with lower toxicity than TSA in HEK 293 cells, indicating that HDAC6-selective inhibitors M344 have potential as drug candidates for HIV-1 eradication. More recently, Sluis-Cremer lab has also reported that HDAC inhibition M344 can induce HIV-1 expression in the J89GFP cells [73].

Following HIV-1 binding and entry, the viral genome has to be reverse transcribed into DNA, transported into the nucleus and integrated into cellular genomic DNA and packaged into chromatin [74]. Verdin and his colleagues showed that the chromatin structure of the HIV LTR contained two well-ordered nucleosomes called Nuc-0 and Nuc-1 [75]. Nuc-0 is found just upstream of the enhancer (−415 to −255), and Nuc-1 is near the viral RNA start site. In order to determine if the reactivation of latent HIV-1 induced by M344 in latently infected cells was due to acetylation of histone at HIV-1 LTR, ChIP assays were performed. Our results showed that M344 increased acetylation of histone H3 (7.4-fold) and histone H4 (16.7-fold) at the nuc-1 region of HIV-1 LTR, which is associated with HIV transcription in A7 cells. This is consistent with a number of studies reporting that the reactivation of HIV transcription requires histone acetylation and remodeling of the critical Nuc-1 by SWI/SNF [47], [76]–[78]. These observations suggest that the acetylation level of histone at the nuc-1 region of the HIV-1 LTR is a key element regulating HIV-1 transcription.

Several studies provide evidence that the presence of histone deacetylases (HDACs) at the HIV LTR is strongly correlated with transcriptional repression leading to latency. Margolis and his colleagues demonstrated that the transcription factor YY1 can act as a repressor of HIV transcription by recruiting HDAC-1 to the provirus [19]. Later studies demonstrated that NF-kB p50 homodimers, CBF-1, AP-4, CTIP2, Sp1, and c-Myc could also recruit HDAC1 [21], [22], [79]–[82]. HDAC2 and 3 can also occupy a site at the HIV LTR and may play a role in the repression of LTR expression [80], [83]. More recently, Sluis-Cremer lab has also demonstrates that HDAC3 resides at the HIV-1 LTR in J89GFP cells and that potent inhibition of HDAC3 may be important for reactivation of latent HIV-1 [73]. It should also be noted that HDAC6 inhibition alone has little effect on HIV LTR expression, as demonstrated in the J89 Jurkat T cell model of HIV latency by an inhibitor selective for HDAC6 (MRK 10) [49], and HDAC6 does not appear to act directly at the HIV LTR [84]. Our data show that HDAC6-selective inhibitors M344 alone were effective in inducing HIV-1 LTR expression in J-Lat Clone A7 cells, and this was associated with significant change in histone H3 and H4 acetylation levels at the HIV-1 LTR promoter. We thus further investigated the localization of HDAC6 and the association of HDAC6 with the HIV-1 LTR promoter during latency in J-Lat cells. Our results confirmed that HDAC6 is present in both the cytoplasmic and nuclear in J-Lat cells, but not occupy to the HIV-1 LTR promoter, indicating that HDAC6 is not directly involved in repressing HIV-1 LTR promoter activity. Zhang *et al* reported that NF-κB p50 and p65 cooperate with HDAC6 to repress transcription of the Ht-Kt-ATPase gene [85]. Girdwood D, *et al* reported that HDAC6 can binds to a domain of the HAT p300 leading to repression of its transcriptional activities [86]. Collectively, we speculate that HDAC6 is not recruited to the HIV-1 LTR promoter, but HDAC6-selective inhibitors M344 can exhibit excellent activity against HDAC6, which will reduce its effect on NF-κB or HAT p300 transcriptional repression. These in turn recruit the cellular histone acetyltransferase p300, driving localized histone acetylation and promoting transcriptional initiation. The mechanisms by which HDAC6-selective inhibitors M344 reactivated latent HIV-1 requires further investigation.

HIV-1 gene expression is strongly dependent on host cell transcription factors. The transcription factor NF-κB plays a central role in the activation pathway of the HIV-1 provirus. Various studies have reported that the 5′LTR of HIV-1 contains several DNA-binding sites for various cellular transcription factors, including Sp1 and NF-κB binding sites, which are required for HIV-1 replication [87], [88], whereas other sites, such as NFAT, LEF-1,COUP-TF, Ets1, USF, and AP-1 binding sites, enhance transcription without being indispensable. To determine whether activation of NF-κB was involved in M344-mediated activation of the latent HIV LTR in the A7 cell model of HIV latency, we first examined the ability of M344 to activate various signaling pathways using reporter plasmids containing either the wild type HIV-1 LTR, the LTR lacking the two κB enhancers, the LTR lacking the AP-1 enhancers, or the LTR lacking the Sp1 enhancers. We observed that M344 effectively activated the wild type HIV-1 LTR-luciferase reporters, reporters with LTR lacking AP-1 or Sp1 enhancers, but displayed no stimulatory effects on the LTR lacking the κB enhancers reporter constructs, indicating that HIV-1 reactivation induced by M344 involves NF-κB signaling pathways. To strengthen this point, we test whether HIV-1 reactivation in latently infected cells induced by M344 is blocked by a NF-κB inhibitor. Several groups have reported that aspirin can inhibit NF-κB activation induced by TNF-α and some other agents through preventing the phosphorylation and degradation of IκBα and nuclear translocation of NF-κB [60], [61], [89], [90]. It have been confirmed that prostratin-mediate activation of the latent HIV LTR by NF-κB signaling pathway [44]–[46]. For this reason, we choose aspirin as the inhibitory agent of NF-κB signaling pathway, TNF-α and prostratin as the inducer of NF-κB in this experiment. We found that pretreatment of J-Lat Clone A7 cells with aspirin can prevent M344, TNF-α or prostratin-induced HIV-1 reactivation, which further supports the findings that M344 activates the HIV-1 LTR through induction of NF-κB signaling pathway. Since HIV-1 reactivation induced by M344 involves NF-κB signaling pathways, it was important to confirm that RelA proteins were transferred to the nucleus and directly recruited to the HIV-1 LTR *in vivo* following M344 stimulation. Using immunofluorescence staining, we observed that M344 induced the translocation of p65 into the nucleus. Using chromatin immunoprecipitation assays, we observed that M344 stimulation promoted rapid recruitment of RelA to the HIV LTR. These observations suggest that reactivation of latent HIV-1 induced by M344 in latently infected cells involves the NF-κB pathway. In supporting this view, a recent study by Li *et al.* reported that M344 treatment could markedly increase the levels of NF-κB activation, indicating that M344 is a potent activator of NF-κB transcription factor [59].

We evaluated whether HDAC6-selective inhibitor M344 could act synergistically with TNF-α, a cytokine that activates HIV-1 transcription through NF-κB (p65/p50) induction, prostratin, the non-tumor-promoting phorbol ester showing a lack of tumor promotion and an ability to block viral proliferation but also an ability to induce latent proviral expression [43]–[45], and 5-Aza, a small molecule inhibitor of DNA methylation, in J-Lat clones A7 cells. Our results showed cotreatment with prostratin/HDACi to induce HIV-1 expression in a higher proportion of J-Lat clones A7 cells than the drugs alone, indicating that M344 can synergistically reactivated HIV-1 production with prostratin in latency model systems. These results are similar to other reports [91], [92] in which the proportion of J-Lat cells displaying GFP epifluorescence was synergistically increased by prostratin/HDACI cotreatments compared to treatments with the compounds alone. While we did not find any synergistic effects when M344 was used in conjunction with 5-Aza or TNF-α, we did observe an additive effect in inducing HIV-1 LTR expression in J-Lat clones A7 cells. These observations are consistent with Jordan. *et al.* reports that treatment with 5-axa-2-deoxycytidine (azadC), an inhibitor of DNA methylation, had little effect on the fraction of cells induced to transcribe HIV alone or in combination with a histone deacetylase inhibitor [64].

In summary, we have provided strong evidence that HDAC6-selective inhibitor M344 is a potent antagonist of HIV-1 latency, acting by increasing the acetylation of histone H3 and histone H4 at the nuc-1 site of the HIV-1 LTR and inducing NF-κB p65 activation. However, it would be important to extend these observations to a wider population of latent cells from infected patients undergoing antiviral therapy to make M344 potential as drug candidates in antilatency therapies. The present findings demonstrate the important role of histone modifications and NF-κB transcription factors in regulating HIV-1 LTR gene expression and raise the possibility that HDAC6-selective inhibitors M344 have potential as drug candidates in antilatency therapies.

## Materials and Methods

### Cell Culture and Chemical Treatment

J-Lat clones A7 cells, a type of latently infected Jurkat cell encoding GFP as a marker for Tat-driven HIV LTR expression, were kindly provided by the NIH AIDS Research and Reference Reagent Program (Dr. Eric Verdin) [64], [65]. The J-Lat clones A7 cells were grown in RPMI 1640 medium supplemented with 10% fetal bovine serum (Gibco), 100 U/ml penicillin and 100 ug/ml of streptomycin (Invitrogen) at 37°C under 5% CO2. Human embryonic kidney 293 cells (HEK 293) were purchased from the American Type Culture Collection and were grown at 37°C in Dulbecco’s modified Eagle’s medium (DMEM) (Gibco) with 10% fetal bovine serum.

M344, 4-dimethylamino-N-(6-hydroxycarbamoyl-hexyl)-benzamide, was purchased from Alexis Biochemicals (ALX-270-297). Recombinant human TNF-α was purchased from Chemicon International. 5-azacytidine (5-Aza) and TSA was purchased from Sigma (A1287, T8552). Prostratin was purchased from LC laboratories (P-4462). M344, TSA, 5-Aza, and prostratin were dissolved in anhydrous dimethyl sulfoxide (DMSO) to a 100-mM stock solution.

### Visualization of GFP

Expression of GFP as a marker for reactivation of HIV-1 promoter in J-Lat clones A7 cells was observed by fluorescence microscopy. After treatment with M344 or TSA at the indicated concentrations, J-Lat clones A7 cells were viewed using a Nikon fluorescent microscope. All microscope samples were photographed using a Nikon E2 digital camera.

### Flow Cytometry

J-Lat clones A7 cells were washed with phosphate-buffered saline (PBS) and incubated with the indicated concentrations of M344 at different points in time, or pretreated with various concentrations of (0, 2.5, 5 and 10 mM) aspirin for 3 hours and subsequently treated with M344 (100 nM) or TNF-α (10 ng/mL) or prostratin (100 nM) or control medium for 24 hours. Cells were washed and resuspended in PBS containing 2% paraformaldehyde. GFP expression was measured by FACScan (Becton Dickinson FACScan Flow Cytometer) and FACS data were analyzed with FLOWJO software (Tree Star, CA). GFP-associated fluorescence was differentiated from background fluorescence by gating of live cells (10,000 events total) and two-parameter analysis.

### Isolation of Primary CD4^+^ T-lymphocytes

Primary peripheral blood mononuclear cells (PBMCs) were separated from erythrocytes by Ficoll density gradient centrifugation. CD4**^+^** T cells were isolated from PBMCs by negative selection bead sorting (Miltenyi Biotec) according to the manufacturer’s instructions. Briefly, PBMC were resuspended in MACS running buffer at 2×10^8^ cells/ml and labelled with the appropriate negative selection biotin-antibody cocktail for 10 min at 4–8°C. Labelled cells were then diluted to 1 × 10^8^ cells/ml in MACS running buffer and incubated with anti-biotin microbeads for an additional 15 min at 4–8°C. The cells were then washed and resuspended in 500 µl MACS running buffer prior to magnetic cell sorting using an autoMACS (Miltenyi Biotech).

### Propidium Iodide Staining

J-Lat clones A7 cells were mock treated or treated with M344 (200 nM) or TSA (200 nM) for 72 hours. After treatment, the cells were washed twice with ice cold PBS, collected by trypsinization and centrifuged. The pellets were re-suspended in 300 µl cold PBS and 700 µl cold 70% ethanol and incubated at 4°C. After centrifugation, the cells were washed and re-suspended in cold PBS, containing RNase (5 mg/ml) and incubated at 37°C for 30 mins. Finally, propidium iodide (1 mg/ml) was added and incubated in the dark for 10 mins and cells were analyzed using FACS Aria 1 (Becton Dickinson, San Jose, CA).

### Cytotoxicity Assay

HEK 293, J-Lat clones A7 cells, Jurkat T cells and primary CD4**^+^** T cells were treated with or without M344 or TSA for 48 hours. For M344 and TSA, concentrations of 25, 50, 100, 200 and 400 nM were used. Proliferation and viability were measured via MTT assay [49]. In brief, 3-(4, 5-dimethylthiazol-2-yl)-2, 5-diphenyltetrazolium bromide (MTT, Sigma) was placed in solution with PBS (5 mg/ml). After 4 hours of incubation, 50 µl of solubilization solution (20% SDS) was added, and cells were then incubated at 37°C for 16 hours. In this assay, MTT was cleaved to an orange formazan dye by metabolically active cells. The dye was directly quantified using an enzyme-linked immunoabsorbent assay reader at 540 nm. The 50% cytotoxic concentration (CC50) was determined from the dose response curve. All experiments were performed independently at least three times in triplicate per experimental point. In addition, two activators cell viability were also measured using MTT assay at concentrations used to measure synergistic reactivation.

### Transient Transfection and Luciferase Assays

Using the diethylaminoethyl (DEAE)-dextran procedure as previously described [38], J-Lat clones A7 cells were transfected with HIV1-LTR luc (from Dr. Warner C. Greene) [43], HIV1-LTRΔκB luc, HIV1-LTRΔAP-1luc, and HIV1-LTRΔSp1luc (from Dr. Andrew D. Badley) [93]. At 24 hours post-transfection, the cells were treated or mock-treated with M344 (200 nM) or TNF-α (20 ng/ml). At 48 hours post-transfection, cells were lysed and assayed for luciferase activity (Promega). Luciferase activities derived from the HIV-1 LTRs were normalized with respect to protein concentration using the detergent-compatible protein assay (Bio-Rad).

### Immunofluorescence Staining

Immunocytochemistry was performed as described previously [38]. Briefly, J-Lat clones A7 cells were treated or mock-treated with M344 (200 nM) or TSA (200 nM) and/or TNF-α (10 ng/ml). After a 30-minute or 2-hour treatment, cells were centrifuged and fixed for 5 minutes with Immunohistofix (Bio-Rad) at room temperature followed by 6 minutes with 100% methanol at 20°C. After two washes with PBS, the samples were saturated with PBS containing 0.5% gelatin and 0.25% bovine serum albumin for 1 hour and stained for 1 hour with a 1/100 dilution of an anti-human p65 rabbit polyclonal immunoglobulin G (IgG) (sc-7151×, Santa Cruz). The samples were then washed three times with PBS containing 0.2% gelatin and incubated for 1 hour with a 1/200 dilution of the secondary antibody: Alexa-555-coupled goat anti-rabbit IgG (Molecular Probes). The samples were then washed three times in PBS with 0.2% gelatin and mounted for analysis on a Zeiss LSM510 laser scanning confocal microscope.

### Western Blotting

To determine the expression levels of various proteins in J-Lat clones A7 cells following various stimulations, Western blot analysis was performed as described previously [21]. Cells were harvested by trypsinization. They were then lysed on ice in a buffer consisting of 50 mM Tris–HCl, pH 8.0, 150 mM NaCl, 2 mM EDTA, 1 mM DTT, 0.1% SDS, 1% Nonidet P-40, 1 mg/ml leupeptin and soybean trypsin inhibitor, 0.5 mM PMSF for 30 minutes. Approximately 50–150 mg of protein extracts were loaded on a 12% polyacrylamide gel. The proteins were then electroblotted onto nitrocellulose membrane and exposed to a blocking buffer consisting of 5% non-fat dry milk in 1×TBST, i.e. 20 mM Tris–HCl, pH 7.6 containing 0.8% NaCl and 0.1% Tween-20 at room temperature for 1 hour. The membrane was incubated with the primary antibodies in blocking buffer, followed by incubation with second antibodies. Bands were visualized using the ECL Western blotting system. The proteins were electro-transferred onto nitrocellulose (GE Healthcare,USA) and subsequently immunoblotted with primary antibodies, i.e., rabbit anti-human anti-acetyl histone 3 (Ac-H3, Milipore), mouse monoclonal antibodies against Actin (AC-74; Sigma, USA) and appropriate secondary antibodies, i.e., goat anti-mouse immunoglobulin (IgG) (1∶1000) or goat anti-rabbit IgG (1∶1000). Afterward, the proteins of interest were visualized using the ECL chemiluminescence system (Santa Cruz Biotechnology, USA).

### Chromatin Immunoprecipitation

Chromatin immunoprecipitation (ChIP) analysis was performed according to Milipore Company online protocol and the procedure described previously [47], [48], [49], [94]. Briefly, J-Lat clones A7 cells (1×10^7^ cells/100 mm dish) were treated with or without M344 (200 nM) or TSA (200 nM) or TNF-α (10 ng/ml) for 4 hours, then crosslinked with formaldehyde to a final concentration of 1% for 10 min at 37°C. The cells were washed in ice-cold PBS twice, resuspended in sodium dodecyl sulfate (SDS) lysis buffer and incubated for 20 minutes on ice. Lysates were sonicated to produce DNA fragments of an average length of 500–1000 bp. Extracts were then diluted tenfold with immunoprecipitation (IP) dilution buffer. Two hundred microliters of the diluted sample (10%) were used as input controls and 2 ml of diluted sonicated extract for IP. After pre-clearing with Protein G agarose for 30 minutes at 4°C with agitation, appropriate antibodies (anti-acetyl histone 3,anti-acetyl histone 4 [Ac-H3 (Lys 9 and 14), Ac-H4 (Lys 5, 8, 12 and 16), Milipore], anti-HDAC2 [sc-7899×, Santa Cruz], anti-HDAC6 [40971, Active Motif], anti-p50 [sc-7178×, Santa Cruz], anti-p65 [sc-7151×, Santa Cruz] and rabbit preimmune IgG [Milipore]) were incubated overnight at 4°C with rotation. To collect the immune complexes, appropriate Protein G agarose mixtures were added to each reaction mixture and the resulting mixtures were rotated for 2 hours at 4°C. Beads were centrifuged and washed for 5 minutes at 4°C with each of the following: low salt, high salt, LiCl, and Tris-EDTA buffer. The immune complexes were eluted by incubation in elution buffer, and supernatants were isolated and further incubated for 4 hours at 65°C to reverse cross-linking. Input controls were treated in the same manner at this point. After reverse cross-linking, proteinase K was added and the mixture was incubated for 1 hour at 45°C. DNA was deproteinized by phenol-chloroform extraction and ethanol precipitation in the presence of 20 µg of glycogen. DNA was washed in 70% ethanol, dried, and resuspended in 20 µl of Tris-EDTA buffer. For a typical PCR, 2 to 5 µl of the 20 µl total DNA was amplified for 32 to 34 cycles and visualized by ethidium bromide staining of agarose gels. Primers for HIV-1 LTR: LTR-109F (5′-TACAAGGGACTTTCCGCTGG-3′) and LTR+82R (5′-AGCTTTATTGAGGCTTAAGC-3′), LTR+178F (5′- TAGCAGTGGCGCCCGAACAGG -3′) and LTR+268R (5′- GCCTCTTGCCGTGCGCGCTTC-3′). Primers for an insulin-like growth factor binding protein 4 (IGFBP4) promoter: IGFBP4 forward (5′- CTTTCTTGCTGCAAAGTCCC -3′) and reverse (5′- ATGGCCTTCCATGCTACAAG-3′), IGFBP4 non-targeting forward (5′- GCCAGGGACCGGTATAAAG-3′) and reverse (5′-GACGTAGCGGGGGAAGTTAG-3′). Primers for detection of control β-actin DNA, forward (5′-GTCGACAACGGCTCCGGC-3′) and reverse (5′-GGTGTGGTGCCAGATTTTCT-3′). DNA products of ChIP were quantitated by real-time PCR (ABI Prism 7900 Real Time PCR System, USA). To calculate the relative acetylation levels, Phosphor Imager data of the amounts of PCR product obtained for immunoprecipitated chromatin samples were normalized against the amounts of PCR product obtained for input DNA. All values represent the average of at least three independent experiments.

## Supporting Information

Figure S1 TSA exhibits cytotoxicity in J-Lat clones A7. J-Lat clones A7 cells were mock treated or treated with M344 (200 nM) or TSA (200 nM) for 72 hours. PI staining was used to determine DNA content levels. Data represent the means standard deviations of three independent experiments.
(TIF)

Figure S2 Cell viability assays on HEK 293 cells treated with two activators. HEK 293 cells were treated with M344, TNF-α, 5-Aza, or prostratin or the mixture of M344 and TNF-α, 5-Aza, or prostratin at the indicated concentrations for 48 hours, measured by MTT assay. Data represent the means standard deviations of three independent experiments.
(TIF)

Figure S3 Cell viability assays on primary CD4^+^ T cells treated with M344 or TSA. Primary CD4**^+^** T cells were treated with M344 or TSA at the indicated concentrations for 48 hours, and measured by the MTT method. Results are presented as a percentage of the O.D. (P = 550) of untreated controls subtracted for background. Data represent the means standard deviations of three independent experiments.
(TIF)

Figure S4 Inhibitory effect of aspirin on M344 induced activation of the HIV LTR. J-Lat clones A7 cells were pretreated with various concentrations of (0, 2.5, 5 and 10 mM) aspirin for 3 hours and subsequently treated with M344 (100 nM) or TNF-α (10 ng/mL) or prostratin (100 nM) or control medium for 24 hours. The percentage of GFP+ cells in M344 or TNF-α or prostratin stimulated cells in either the absence or the presence of the chemical inhibitors was measured by flow cytometry. Data represent the means standard deviations of three independent experiments.
(TIF)
