# Supplementary figures and images for: Selective Histonedeacetylase Inhibitor M344 Intervenes in HIV-1 Latency through Increasing Histone Acetylation and Activation of NF-kappaB
Source: PLoS One. 2012 Nov 15;7(11):e48832. doi: 10.1371/journal.pone.0048832 (PMC3499534; doi:10.1371/journal.pone.0048832)

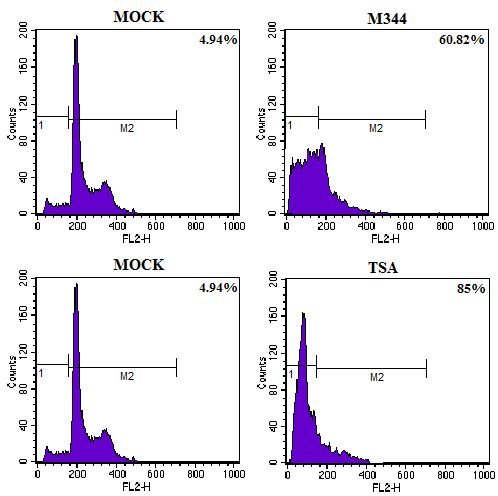

Supplement: Figure S1 — TSA exhibits cytotoxicity in J-Lat clones A7. J-Lat clones A7 cells were mock treated or treated with M344 (200 nM) or TSA (200 nM) for 72 hours. PI staining was used to determine DNA content levels. Data represent the means standard deviations of three independent experiments. (TIF) [file pone.0048832.s001.tif]

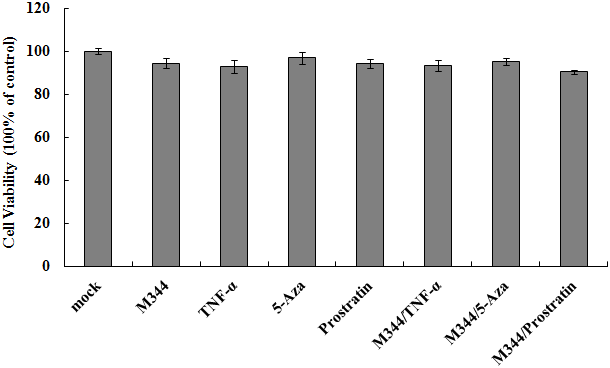

Supplement: Figure S2 — Cell viability assays on HEK 293 cells treated with two activators. HEK 293 cells were treated with M344, TNF-α, 5-Aza, or prostratin or the mixture of M344 and TNF-α, 5-Aza, or prostratin at the indicated concentrations for 48 hours, measured by MTT assay. Data represent the means standard deviations of three independent experiments. (TIF) [file pone.0048832.s002.tif]

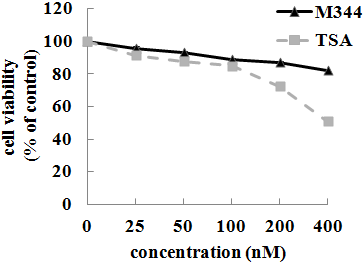

Supplement: Figure S3 — Cell viability assays on primary CD4+ T cells treated with M344 or TSA. Primary CD4+ T cells were treated with M344 or TSA at the indicated concentrations for 48 hours, and measured by the MTT method. Results are presented as a percentage of the O.D. (P = 550) of untreated controls subtracted for background. Data represent the means standard deviations of three independent experiments. (TIF) [file pone.0048832.s003.tif]

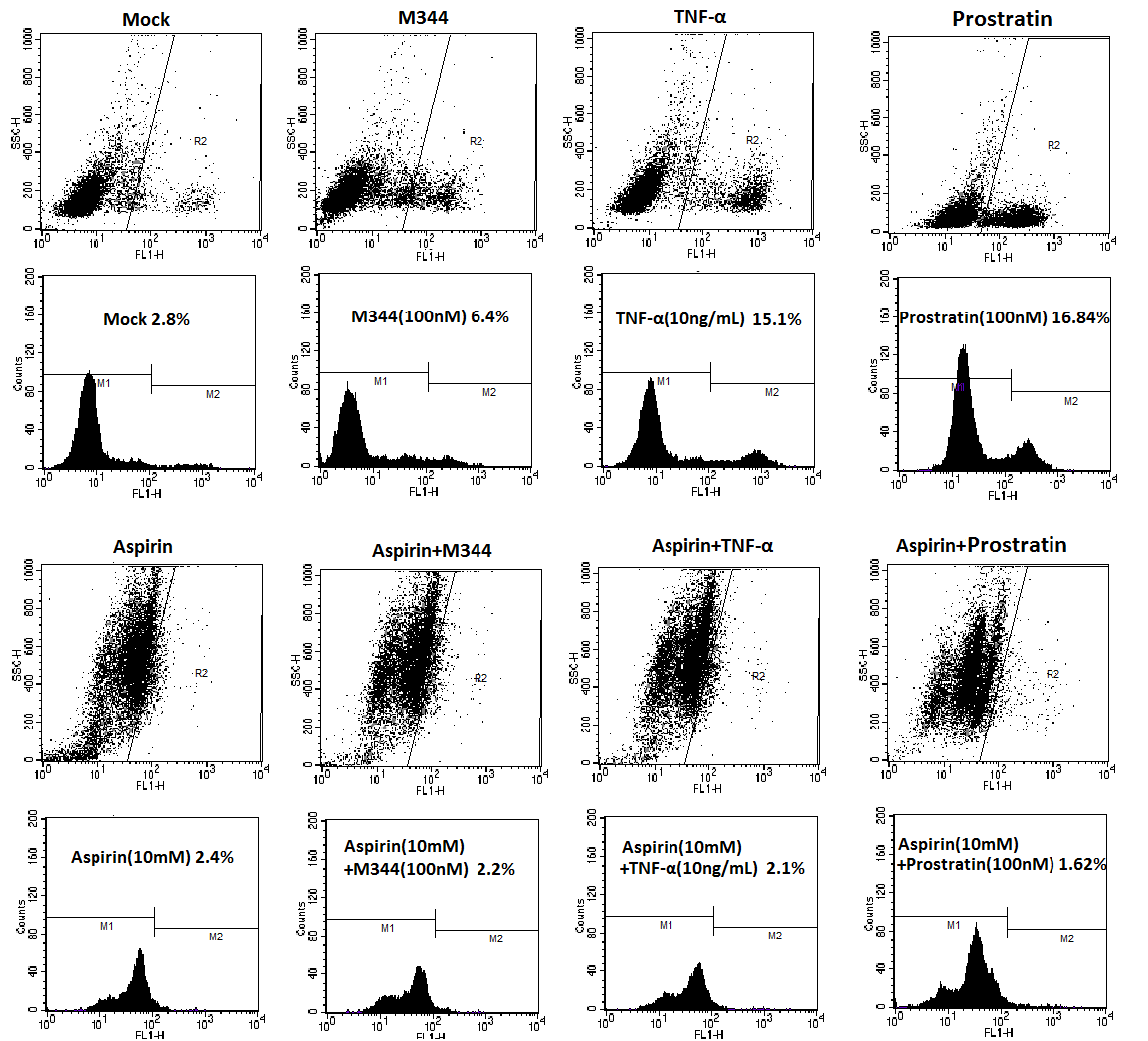

Supplement: Figure S4 — Inhibitory effect of aspirin on M344 induced activation of the HIV LTR. J-Lat clones A7 cells were pretreated with various concentrations of (0, 2.5, 5 and 10 mM) aspirin for 3 hours and subsequently treated with M344 (100 nM) or TNF-α (10 ng/mL) or prostratin (100 nM) or control medium for 24 hours. The percentage of GFP+ cells in M344 or TNF-α or prostratin stimulated cells in either the absence or the presence of the chemical inhibitors was measured by flow cytometry. Data represent the means standard deviations of three independent experiments. (TIF) [file pone.0048832.s004.tif]
